# Moral Mania

**Published:** 1895-08-10

**Authors:** 


### Moral Mania.

While we are not inclined to reckon all crime is simply disease for which the criminal cannot be held responsible, it cannot be denied that sometimes brain disease may take a moral rather than an intellectual form, and the patient be perfectly fit to associate with his fellows, in so far as capacity for ordinary business goes, but totally unfit on account of malicious, dishonest, or murderous proclivities. The point at which irresponsibility comes in to extenuate wickedness is difficult to define in many cases, but when a millionaire pockets a silver spoon when he is dining out, we may reasonably define the act as kleptomania rather than theft. The absence of comprehensible temptation is an obvious reason for regarding an act as morbid rather than criminal, but very few instances can show so clear an explanation. Especially with children it is hard to say where the natural heedlessness of youth degenerates on the one hand into the wilful malice and cruelty of the criminal, and on the other into the instinctive wickedness of the "mind diseased." A case reported by Dr. Samuel Fort, of Ellicott City, Maryland, is almost a perfect type of such mania. A girl who had been dull, stupid, unresponsive, and uncleanly became, under the discipline of an institution for the feeble-minded, comparatively intelligent and decidedly affectionate. But suddenly one day she struck her attendant, for whom she professed great attachment, and in the act her recently-acquired intelligence evaporated, and she relapsed into the state of imbecility that had marked her a year before. Under renewed treatment she advanced again to her old pinnacle, only to fall back again after a similar piece of violence, the awakening of her intelligence seeming to lead only to these fits of passion, and be destroyed in them.

Another case, a boy under the observation of Dr. Martin Barr, superintendent of the Pennsylvania Training School for Feeble-minded Children, showed greater ingenuity. He had more apparent intellectual power; he could read and write, could sing, and had some talent for drawing. But he was restless and malicious, and personally indifferent to danger. In his record it is written: "Powers of attention, imitation, and memory good." This he soon began to prove after he came to the school. He saw an epileptic in a fit. and immediately afterwards fell on the floor and tried to imitate the attack. He went in for fits, sometimes indulging in two or three in one day, and on one memorable occasion in ten at intervals of about an hour and a half. At first the ordinary remedies were tried, but the absence of the ordinary symptoms of epilepsy, and the fact that no fit ever took place unless there were spectators present aroused suspicion, and a system of wrapping in wet sheets proved in the long run beneficial, though the habit of simulating spasms still continues. The boy had had genuine epileptic fits and, having been over-indulged on account of these, regarded them as a clever trick by means of which he could get his own way. When he saw that his deceit was found out, he calmly offered to have a spasm if he was given five cents. Dr. Barr gave him the money, and immediately he fell into a realistic spasm. But when he came out of it, he offered to have a better fit for ten cents, saying, "the better the pay, the harder the spasm." It is evident that a lad like this, if let loose on the world, might work endless mischief, and shelter himself more or less by the plea of irresponsibility. Irresponsible he is in the deepest sense, in that he is not susceptible either to personal fear or regard for others; but he is not irresponsible in the sense of not knowing that his acts were evil, and had he come into a court of justice, it would have been difficult for an ordinary judge to say whether he was a criminal or a lunatic.

The cases which come under the notice of doctors are mostly of a pronounced nature; their family have endured their escapades as long as they could before they came to the conclusion that there was some mental flaw, and in milder cases the weakness may show itself only in a sort of dramatic instinct, which makes the most of little ailments in order to arouse sympathy. A pretence of running away from home is very popular with some, and, snugly ensconced in some hiding-place, the hero of the adventure will watch the tears and anxiety of his family, and see search parties sent out to look for him dead and alive. Then, when he thinks the excitement has reached a sufficient height he makes his reappearance, to be wept over and caressed. It is difficult to modify a tendency like this. With most of us life cures any excessive notions we may cherish as to our own importance, and teaches us that we are but units in a multitude, but with the feeble-minded the *ego* is supreme, and if he cannot concentrate attention on himself by legitimate means he will try others. For him home life is bad, because at home he is a creature apart. To feel himself one of a crowd, however, subjected to a common, not a special discipline, has a sobering influence upon him. Manual work is very useful for such cases, partly because it is so difficult to pretend either to have done it or to be unable to do it; and partly because it withdraws the mind from contemplating itself. In some cases, distinct moral training answers well. You may get a feeble-minded child who can grasp the distinction between right and wrong. With another affection for parent or teacher may control the egoistic weakness. With those of a lower grade, the mere knowledge that no one is moved by their tricks may act as a deterrent. But with all, the distraction brought about by physical work is helpful, and under judicious control the morbid vanity may be developed into a healthy desire to excel. But there will always be the danger of the corresponding vice, a malicious hatred of all who are more accomplished, with perhaps an attempt to destroy better work, and injure those who do it. Very sad is it to see these nursery tricks in grown men and women; but it is just such notions as justice and fair play which it is most difficult to inculcate into the feeble-minded. They can grasp many of the more showy virtues more easily, and in them you may find the most reckless generosity, the promptest self-sacrifice, where you will often look in vain for honesty and ordinary consideration for others in the details of daily life.

